# Different Diagnostic Criteria for Determining the Prevalence of Sarcopenia in Older Adults: A Systematic Review

**DOI:** 10.3390/jcm13092520

**Published:** 2024-04-25

**Authors:** Blanca Pedauyé-Rueda, Pablo García-Fernández, Luis Maicas-Pérez, José Luis Maté-Muñoz, Juan Hernández-Lougedo

**Affiliations:** 1 HM Faculty of Health Sciences, Camilo José Cela University, 28692 Madrid, Spain; blanca.pedauye@ucjc.edu (B.P.-R.); jlougedo@ucjc.edu (J.H.-L.); 2 Department of Radiology, Rehabilitation and Physiotherapy, Complutense University of Madrid, 28040 Madrid, Spain; pablga25@ucm.es; 3 Atlético de Madrid Foundation, 28004 Madrid, Spain; lmaicas@atleticodemadrid.com; 4 Faculty of Health Sciences, Universidad Internacional de la Rioja, 26006 Logroño, Spain

**Keywords:** sarcopenia, diagnosis, consensus, prevalence, assessment, aged, aging and older

## Abstract

**Background**: Sarcopenia is defined as a loss of muscle mass, strength, and physical function associated with aging. It is due to a combination of genetic, environmental, and physiological factors. It is also associated with an increased risk of health problems. Since there are many different researchers in the field, with their own algorithms and cut-off points, there is no single criterion for diagnosis. This review aims to compare the prevalence of sarcopenia according to these different diagnostic criteria in older adult populations by age group and sex. **Methods**: Different databases were searched: Web of Science, Pubmed, Dialnet, Scopus, and Cochrane. The keywords used were “sarcopenia”, “diagnosis”, “prevalence”, “assessment”, “aged”, “aging” and “older”. Studies conducted in a population aged ≥65 assessing the prevalence of sarcopenia were selected. **Results**: Nineteen articles met the inclusion criteria, with a total of 33,515 subjects, 38.08% female and 61.42% male, at a mean age of 74.52. The diagnostic algorithms used were 52.63% AWGS2, 21.05% EWGSOP2, 10.53% AWGS1 and EWGS1, and 5.26% FNIH. Prevalence ranged from 1.7% to 37.47%, but was higher in males and increased with age. **Conclusions**: The prevalence of sarcopenia varies depending on the diagnostic algorithm used, but it increases with age and is higher in men. The EWGSOP2 and AWGS2 are the most used diagnostic criteria and measure the same variables but have different cut-off points. Of these two diagnostic algorithms, the one with the highest prevalence of sarcopenia and severe sarcopenia is the AWGS2. These differences may be due to the use of different tools and cut-off points. Therefore, a universal diagnostic criterion should be developed to allow early diagnosis of sarcopenia.

## 1. Introduction

Sarcopenia is a condition characterized by a gradual decline in muscle mass, strength, and physical performance that increases as individuals age [1]. Sarcopenia has been included in the International Classification of Diseases and Related Health Problems by the World Health Organization since 2016, with the diagnosis code ICD-10-MC (M62.84) [2]. Sarcopenia occurs mainly in older adults because from approximately the fifth decade of age, the annual loss of muscle mass is 0.8% and the loss of strength is 3% [3,4].

Sarcopenia is influenced by a variety of genetic, physiological, and environmental factors, and preventing its development in older age is difficult. It is important to consider the various stages of sarcopenia, which range from probable sarcopenia to severe sarcopenia [5]. It is linked to a higher likelihood of health issues, such as disability, functional limitation, and functional decline [6], increased risk of falls [7], longer hospital stays [8] and increased risk of mortality [9].

Despite the seriousness of the condition, there is no universal diagnostic standard. Various working groups have developed different diagnostic methods and cut-off points. These groups use a range of factors including muscle mass, grip strength, and gait speed as primary indicators. Some widely adopted definitions have been formulated by the European Working Group on Sarcopenia in Older People (EWGSOP), the Asian Working Group for Sarcopenia (AWGS), and the Foundation for the National Institutes of Health (FNIH). There are also others such as the International Working Group for Sarcopenia (IWGS) and the Sarcopenia Definitions and Outcomes Consortium (SDOC). Cut-off points and assessment tools used to diagnose sarcopenia vary among the groups [10].

It should be noted that the first step in the diagnosis of sarcopenia is case finding. While the European consensus (EWGSOP) only uses the SARC-F questionnaire, the Asian consensus (AWGS) employs two additional techniques, namely the SARC-CalF survey and lower limb circumference. The next stage identified in the diagnostic algorithm involves muscular endurance through handgrip strength assessment using a handheld dynamometer to measure maximal grip force exertion and a 5-time chair-stand test to assess the time needed for a participant to complete five consecutive chair stand-ups without using their arms [11]. The EWGSOP diagnostic tree requires reliable results in both these tests before advancing to the third step, whereas only one is needed in the case of the Asian Consensus. Dual Energy X-ray Absorptiometry is used to assess the quantity and quality of muscle in confirming sarcopenia (DEXA) or electrical bioimpedance (BIA) [12,13]. Finally, to assess the severity of sarcopenia, both groups conduct tests to evaluate physical performance. One such test involves measuring walking speed by timing the subject as they walk a distance of 4 m; another is the Timed Up and Go (TUG), which consists of getting up from the chair, walking 3 m, going around a cone and sitting back in the chair. The Short Physical Performance Battery (SPPB) consists of a series of assessments, such as the sit-to-stand test, TUG, and a three-position balance test (feet together, tandem and semi-tandem), which are scored based on how long the position can be maintained. Other organizations have established specific criteria for diagnosing sarcopenia based on muscle mass, grip strength, and gait speed, including the IWGS and the SDOC [12,14]. The initial set establishes cut-off points for gait speed and appendicular muscle mass index measured by DEXA, whereas the second category identifies sarcopenia in cases of diminished gait speed and grip strength [14]. In contrast to the EWGSOP and AWGS, neither of these two working groups assesses the diagnosis of severe sarcopenia [12,14]. Table 1 shows the differences in the diagnosis of sarcopenia between the different working groups.

A research study assessed the occurrence of sarcopenia using the EWGSOP and FNIH diagnostic criteria. The findings revealed prevalences of 5.3% and 0.5% in men, and 13.3% and 1.8% in elderly women, respectively [15]. In other studies, conducted on similar populations, the prevalence varies up to 40% depending on the definition applied [16,17].

Due to the globally rising population of elderly individuals, it is crucial to conduct assessments to identify sarcopenia and prevent its onset. Since scientific studies indicate significant variations in tests and diagnostic methods among different research groups, this review aims to compare the prevalence of sarcopenia using diverse diagnostic criteria in older adult populations in terms of age and gender.

## 2. Materials and Methods

This study was developed following the Preferred Reporting Items for Systematic Reviews and Meta-Analyses (PRISMA) guidelines [18] and the suggestions of the Cochrane Collaboration Manual [19].

### 2.1. Search Strategy

A systematic search was conducted up to November 2023 in the following databases: Web of Science (WOS), PubMed, Cochrane, Scopus and Dialnet, to ensure the most extensive results. Incomplete articles were identified, and missing information was obtained by contacting the authors via email. After a preliminary search, a second search was conducted using controlled and natural language revised by a health sciences librarian across all the mentioned databases. See Table 2 for an example of our search strategy.

### 2.2. Article Selection

We included scientific articles that met the following inclusion criteria: (a) articles published in the last seven years, (b) articles in English or Spanish, (c) articles in adults aged ≥65, (d) observational and cross-sectional studies, (e) studies relating to the diagnosis and prevalence of sarcopenia. Exclusion criteria were: (i) use of animals as a sample and (ii) systematic reviews. Assessment eligibility was determined using the online software CADIMA version 2.2.3, Julius Kühn Institute, Erwin-Baur-Str. 27, 06484 Quedlinburg, Germany (www.cadima.info (accessed on 7 December 2023)). Following the PRISMA flowchart, two reviewers assessed the relevance of studies on the same platform in an independent, standardized, and unblinded manner (BPR, JHL). CADIMA also allowed for an interobserver consistency study.

### 2.3. Data Analysis

An extraction sheet was modified from the Cochrane template “Data Collection Form for Intervention Reviews: RCTs and non-RCTs”. Duplicate articles were removed with the assistance of the reference management tool Mendeley (version 2.100). Data from the included studies were extracted by one investigator (BPR) and reviewed by a second investigator (JHL). Discrepancies were then resolved by a third investigator (PGF). We present the data relevant to the objectives of our study in a systematic and synthesized way (tabular format).

### 2.4. Evaluation of the Quality and Risk of Bias of the Studies

To assess the risk of bias and methodological quality of the studies, we used the Newcastle–Ottawa Scale (NOS) adapted for cross-sectional studies [20]. The NOS scale consists of six items that assess sample representativeness, sample size, non-responders, determination of exposure (items 1–4), comparability in different groups (item 5), and evaluation and analysis of the results (item 6). The maximum score is 10, and the points are distributed as follows: selection (items 1–4) 5 points, comparability (item 5) 2 points, and results 3 points (item 6). Studies with a score of 0–5 points are considered at a high risk of bias, while scores of 6–10 indicate low risk.

### 2.5. List of Working Groups for the Diagnosis of Sarcopenia

Table 3 shows the working groups created to establish algorithms for the diagnosis of sarcopenia, and the year of each group’s consensus publication.

## 3. Results

### 3.1. Selection of the Studies

A total of 21,764 articles were found across various databases. After removing duplicate entries, this number was reduced to 11,889. To narrow down the search, inclusion criteria such as publication dates (2017–2023) and cross-sectional observational studies in both English and Spanish were applied using the automatic lateral filters in Pubmed and WOS. At the end of this process, 870 articles remained. After reading titles and abstracts, 851 were discarded since they were not related to the objectives of the review. Ultimately, 19 articles were included in the review.

**Figure 1:**
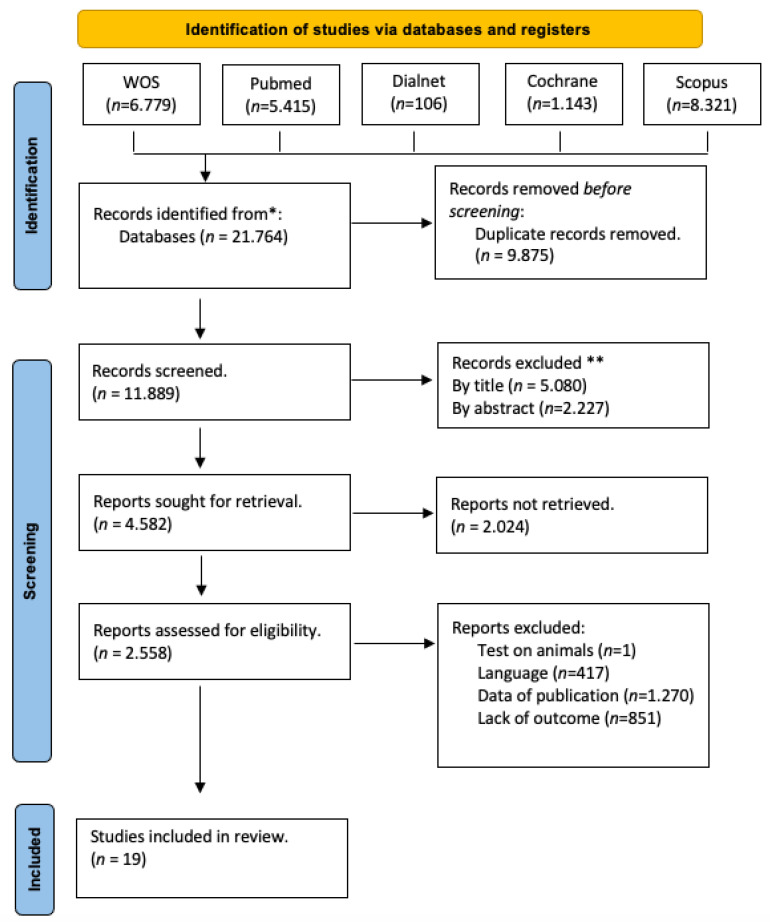
Flow diagram according to the PRISMA declaration. * = The number of articles found in each database is indicated; ** = No automation tools were used.

See Figure 1 for the selection process, conducted in accordance with the PRISMA 2020 declaration.

### 3.2. Individual Study Results

Table 4 shows the results obtained in each of the studies, analyzed individually.

### 3.3. Participants, Year of Publication, and Diagnostic Algorithms

Our research involves a study population of 33,515, 12,763 (38.08%) women and 20,588 (61.42%) men. Of the total number of articles included in the review, 5.26% were published in 2017, 21.05% in 2019, 10.53% in 2020, 10.53% in 2021, 42.11% in 2022 and 10.53% in 2023. The mean age of the participants was 74.52, ranging between 60 and 100. The youngest mean age of the participants was 67.7 ± 6.4 years old [21] and the oldest was 85.5 ± 0.4 years old [39]. The number of subjects in each of the studies was diverse, with the smallest sample being 161 females [34] and the largest 9170 men [24].

All studies included in the analysis assessed the occurrence of sarcopenia, with four specifically measuring severe sarcopenia. In terms of diagnostic methods employed, more than half (52.63%) employed the criteria developed by AWGS2, followed by 21.05% using EWGSOP2 criteria. Those outlined by AWGS1 (10.53%), EWGSOP1 (10.53%), and FNIH (5.26%) were used in smaller proportions.

The research was conducted globally with 31.58% of the studies involving participants from China, 15.79% including Japanese populations, 21.05% focusing on South Korean cohorts, 10.53% of the studies conducted with Brazilian populations, and 5.26% with individuals from Australia, Thailand, and the United States. One study collected data from multiple countries including the United States, Sweden, England, and Belgium. However, in some of the studies reviewed, the European diagnostic algorithm was used in Asian or no Asian populations [24,27,28,30,34,38,39].

### 3.4. Prevalence according to Diagnostic Algorithm, Age Group, and Sex in the Included Studies

See Table 5 for the average occurrence rate for each age category, categorized by gender and based on the various diagnostic methods used in the studies included.

### 3.5. Risk of Bias and Methodological Quality of Included Studies

The mean score obtained on the NOS scale was 7.84 points. Among the studies, 5.26% presented a high risk of bias, corresponding to the study by Moreira et al. conducted in 2019 [28], with 94.74% at a low risk of bias. In the section for statistical evaluation and analysis, all studies reached the highest score. In terms of comparability, 47.37% of the studies reached the maximum score while 47.38% obtained half of the total score. In factors related to selection, there was more variation in scores among the studies. Out of a possible five points in this section, 36.84% of articles scored 4 points, 31.58% scored 3 points and 15.79% only 2 points. It should be noted here that items associated with sample size and non-responding subjects pose higher risks for bias in these studies. See Table 6 for details of each individual study.

## 4. Discussion

Since 2016, there have been no comprehensive reviews conducted on the frequency of sarcopenia in older adults that distinguish between various diagnostic methods. As the prevalence of sarcopenia is a serious concern for health institutions due to the increase in the elderly population worldwide, this review aims to compare the prevalence of sarcopenia according to different diagnostic criteria in older adult populations according to age group and sex.

In the 19 studies analyzed, AGWS2 was the most frequently used diagnostic algorithm (52.63%), followed by EWGSOP2 (21.05%), AWGS1 (10.53%), and EWGSOP1 (10.53%). FNIH and SDOC each represented 5.26% of the total usage. All these studies were observational, focusing on determining the prevalence of sarcopenia. The average methodological quality assessed using the NOS scale was 7.83 points, indicating a low risk of bias and high quality overall. Only one study showed lower methodological quality (5 points) due to a higher risk of bias in sample selection [28].

Sarcopenia is emerging as a significant health concern with the global aging population on the rise. Our research indicates a substantial variation in prevalence depending on the diagnostic approach employed. It is crucial to establish unified criteria to enable accurate comparison of sarcopenia prevalence across diverse populations. Accurate diagnosis is essential for early detection and prevention or intervention against disease progression.

When examining the consensus developed by the Asian and European working groups, it is evident that the diagnostic criteria of the Asian group are more stringent. They set higher thresholds for the assessed parameters, while fewer tests are needed to confirm sarcopenia [2,4]. The IWGS requires a higher threshold for gait speed but assesses fewer variables than the AWGS and EWGSOP in diagnosing sarcopenia [3].

### 4.1. Asian Working Group (AWGS)

When examining the variations in sarcopenia prevalence as per the Asian working group, it is important to note that there are two distinct consensuses: one developed in 2014 and another in 2019. AWGS2 considers a broader range of factors, sets higher cut-off points for grip strength and gait speed, and introduces the concept of severe sarcopenia.

In the studies employing the AWGS1 diagnostic algorithm, the mean age was 74 with a prevalence of 10.1%. It was higher in men (14.7%) than in women (8.3%) [22,25,30]. Only one of the three research projects categorized the participants based on age and gender. According to that study, the occurrence rate was greater in males across all age categories [25]. As age increased by five years, the prevalence increased by 8.76% in men and 3.83% in women [25]. However, even though they all used the same diagnostic algorithm, and the subjects had a similar mean age (70–75), the prevalence ranged from 6.6% to 6.7% in men and from 3.83% [22] to 12.2% [25] in women. The variations in prevalence could be attributed to disparities in the gender ratio and racial composition of participants across studies.

The AWGS2 algorithm is the most used in the studies analyzed, in more than 50%. The mean age of the subjects was 73.1, and the prevalence was 24.64% [21,23,26,29,31,32,33,35,37]. When the AWGS2 algorithm was used, similar to the case with AWGS1, a higher prevalence of 26.73% was observed in men compared to 24.66% in women.

In addition to sarcopenia, two studies identified the existence of severe sarcopenia and obesity-related sarcopenia [33,35]. The prevalence of both is also higher in men than in women. The prevalence of severe sarcopenia was 11.5% in men and 8.5% in women [33] and in sarcopenic obesity the prevalence was 13.94% and 7.14% in men and women, respectively [35]. In all research examining the occurrence of sarcopenia across different age groups, it was observed that the likelihood of positive diagnoses for sarcopenia increases with advanced age. In the age cohort between 65 and 80, there was an average prevalence increase of 7.08% every five years. This rate rises to 15.88% [23,32,35,37], in line with those studies using the AWGS1 algorithm, where while the mean age ranges from 67.7 to 77.8, the prevalence ranges from 14.73% [21] to 43.4% [23]. The wide variation in the prevalence reported when employing this diagnostic algorithm may be attributed to the diverse range of assessments used to confirm sarcopenia, including tests for grip strength, gait speed, physical performance, and tools to determine muscle mass [40].

It is also important to consider that when evaluating muscle strength in this population, parameters such as grip strength or physical performance (such as gait speed, SPPB, or 5-time chair-stand test) may be used. A study found a higher prevalence difference based on the tests conducted, with gait speed at 40.97% and grip strength at 37.63% [31].

### 4.2. European Working Group (EWGSOP)

Like the Asian working group, the European working group also has two consensuses: one dated 2010 and the second 2019. The key variances between them include the incorporation of the SARC-F questionnaire and the 5-time-sit-to-stand test, as well as lower threshold values for grip strength and musculoskeletal mass to confirm sarcopenia. For diagnosing severe sarcopenia, it includes the TUG and SPPB tests [2].

Two out of the nineteen research papers analyzed in the review implemented the EWGSOP1 diagnostic algorithm. Both studies found a similar prevalence of the condition in men (9% and 20.91%) and women (8% and 20.59%) [28,39]. However, the prevalence of severe sarcopenia was 4% higher in women than in men [28]. This prevalence tends to rise with age, but there is significant variation in the prevalence rates across different age ranges. It was 6.3% for individuals aged 65–74 and increased to 55.67% for those aged 75–84 [28]. Regardless of gender, there is a notable difference in the occurrence rates observed in both studies. This contrast may be attributed to the variance in average age, which was 76.6 ± 6.9 and 85.5 ± 0.4 for each study respectively [28,39].

The prevalence of sarcopenia has been widely researched in diverse studies on different ethnicities according to sex, age, or both using the diagnostic criteria developed by EWGSOP2 [24,27,30,34,38], the second-most-used diagnostic algorithm in the included studies, after AWGS2. However, since four out of the five studies using this algorithm did not jointly use 5-chair-sit-to-stand and grip strength [24,27,34,38], meaning that the diagnostic algorithm was not applied correctly, this could cause the prevalence estimate to be lower. The mean age of the subjects included in the studies using this diagnostic algorithm was 73.94 and the prevalence was 5.95% [24,27,30,34,38]. In relation to sex, as with the studies using the AWGS1 and AWGS2 algorithms, it was higher in men (8.3%) than in women (4.93%).

Two research studies examined the frequency of occurrence based on gender and age bracket [27,38]. In both studies, the frequency rose with advancing age, showing an average increase of 1.68% every five years between ages 65 and 80 [27,38]. However, beyond the age of 80, the prevalence increased by 11.04% every five years [27,38]. This was reported more in males across all age ranges, except for the 75–79 age bracket, where it stood at 8% among women [27].

In a separate research study that examined the frequency of sarcopenia based on different tests for evaluating muscle strength, it was found that the prevalence was 3.8% greater when using the 5-time-chair-to-stand test (6.5%) compared to the grip strength assessment (2.7%) [34].

### 4.3. SDOC and FNIH

These two research groups determine sarcopenia by examining a reduced number of factors compared to the aforementioned sources. The SDOC examines grip strength and gait speed, while the FNIH assesses appendicular muscle mass and gait speed. This approach introduces potential inaccuracies in diagnosis, which may explain why these methods were used in only two studies included in this review. In the first study using the FNIH-developed algorithm, sarcopenia was found to have an overall prevalence of 37.43%, with rates of 39.41% in men and 36.45% in women [36]. However, in the second study using the algorithm developed by the SDOC, the total prevalence was 1.7% and the average increase in prevalence was 1.57% every five years [24]. These differences in prevalence may be due to lower cut-off points for grip strength in FNIH than in SDOC [41,42]. However, the difference in sarcopenia prevalence within the same population was only 0.6% higher when employing the SDOC diagnostic criterion compared to the EWGSOP2 [24].

After analyzing the different studies, we can observe that the prevalence of sarcopenia in a population of similar age using different algorithms varies in a range between 1.7% (SDOC) and 37.43% (FNHI). In men, it varies between 8.3% (EWGSOP2) and 39.41% (SDOC), and in women between 4.93% (EWGSOP2) and 36.45% (SDOC). These differences in prevalence may be because the number of subjects was nine times higher in the study applying the algorithm developed by the FNIH [36] than in that using the algorithm developed by the SDOC [24]. In one study examining the general occurrence of sarcopenia, it was discovered that it affected 10% of both male and female individuals [43]. There was a higher prevalence observed in non-Asian participants when compared to Asians [43]. Analyzing the prevalence of sarcopenia in all the studies included in the review, it is observed to increase with age, regardless of the diagnostic method used. The prevalence was 5.32% in the 65 to 69 age group and 27% in individuals aged ≥85. This higher prevalence of sarcopenia might be attributed to the decline in muscle mass and strength as people get older, particularly among those with low levels of physical activity. In addition, the number of criteria needed to confirm sarcopenia is lower compared to other diagnostic criteria [2,4,12,14].

When classifying subjects by both age group and gender (Table 5), men consistently exhibit a higher prevalence regardless of the diagnostic approach employed. There are only two exceptions: In the 80–84 age group, according to the EWGSOP2 algorithm and AWGS2 criteria, women have a notably higher prevalence at 1.75% and 4.89%, respectively. In addition to age and gender, variations in the incidence of sarcopenia may arise from the different factors and assessment tools employed by different research groups in their diagnostic methods.

### 4.4. Diagnostic Algorithm Sections

The SARC-F questionnaire is widely used to identify sarcopenia cases and consists of five elements that evaluate muscle strength, the need for walking assistance, the ability to rise from a chair, stair-climbing difficulty, and the frequency of falls within the previous year [44]. In both the diagnostic criteria for sarcopenia in Europe and Asia, the initial step is similar. However, the Asian criteria incorporate other aspects that may indicate probable sarcopenia, such as measurements related to muscle strength and function using the SARC-CalF questionnaire, along with consideration of medical conditions like COPD, diabetes mellitus, heart failure, depression, cognitive impairment, malnutrition, or continued weight loss. The SARC-F questionnaire has been validated for assessment purposes; it includes open-ended questions which may often yield negative results (<4 points) when other assessed variables point towards a positive diagnosis of sarcopenia. This leads to numerous undetected cases with a positive diagnosis potentially resulting in increased health repercussions associated with sarcopenia, including higher morbidity rates or longer hospital stays, highlighting one significant contrast between EWGSOP2 and AWGS2 guidelines [2,40]. Variations in tests/conditions for case detection may contribute to the observed higher mean prevalence when utilizing AWGS2’s diagnostic algorithm compared to EWGSOP2.

In relation to muscle mass, computed tomography and magnetic resonance imaging are considered the most reliable methods for measuring muscle mass. However, their limited accessibility and high cost make them less commonly used in research [45,46,47]. AWGS sets varying thresholds based on the measuring instrument, including DEXA and BIA, whereas EWGSOP only defines cut-off points for use with BIA. It has been observed that DEXA yields inconsistent results since it is unable to differentiate between other tissues or components such as connective tissue or water when assessing muscle mass [14,48,49,50]. It has been determined that employing deuterium-labeled creatine (D3-Cr) is a precise method for assessing skeletal muscle mass [14,48,49,50].

Prevalence estimates obtained through DEXA measurements vary widely, with rates ranging from 2.2% to 95% for men and from 0.1% to 33.9% for women [51]. BIA-produced prevalence figures ranged between 6.2% and 85.4% for men and between 14.1% and 23.6% for women, while computed tomography showed a range of prevalence between 14.1% and 55.9% [51].

This study demonstrates notable differences in prevalence based on the choice of measurement tool [51]. It is also crucial to consider the specific anthropometric variable used for assessing muscle mass. For instance, when using the appendicular muscle mass index, prevalence varies from 0.0% to 56.7% among men and from 0.0% to 33.9% among women [51]. These discrepancies may be attributed to population-specific factors like physical activity levels, dietary habits, or cultural traits [51]. A study that analyzed the differences between Asian and non-Asian subjects concluded that younger Asians have a lower amount of muscle mass, engage in more physical activity and have a different diet [43]. This could potentially explain why the Asian group established different cut-off points, leading to a higher prevalence of sarcopenia when this diagnostic algorithm is applied.

According to the diagnostic algorithms developed by all working groups, the assessment of muscle strength is what allows us to confirm sarcopenia. A hand dynamometer is commonly used to measure grip strength. EWGSOP introduces another evaluation method—the 5-time chair-stand test. However, this test yields less reliable and valid data due to its reliance on timing, which may be influenced by human bias and error. AWGS2 also incorporates a 5-time chair-stand test for assessing physical performance in elderly individuals [52,53]. Low grip strength has been linked to higher levels of functional limitations, extended hospital stays, and elevated mortality rates [54,55]. It is documented that the use of a hand-held dynamometer may be the most suitable method for evaluating muscle strength in individuals diagnosed with sarcopenia [56].

This is another factor in which the Asian and European groups show differences, and it is particularly important for confirming the diagnosis. According to the EWGSOP2, positive results for grip strength and 5-time chair-stand tests are necessary for the confirmation of sarcopenia. AWGS2 allows for a broader range of possibilities, as it only requires one of the tests to be positive (grip strength test, 5-time chair-stand test, gait speed) [2,40]. This could be a factor contributing to the higher prevalence observed with AWGS2 compared to EWGSOP2, regardless of the individuals’ gender, ethnicity, and age. When comparing two studies with Chinese populations of the same age and gender, differing only in the diagnostic algorithm employed (EWGSOP2 and AWGS2), it is observed that in the 65–69 age group, the prevalence of sarcopenia was higher with the Asian criterion (AWGS2) compared to the European criterion (EWGSOP2), being 6.32% higher in men and 6.46% higher in women. The same pattern is observed in all age groups; in the 70–74 age group, sarcopenia prevalence with AWGS2 was higher by 0.88% in men and 10.83% in women; in the 75–79 age group, prevalence was 12.37% higher in men and 10.38% in women; and finally, in the 85 years or older age group, prevalence was 9.64% higher in men and 23.92% in women. The same trend is observed when comparing two studies with Japanese populations, which classified groups into the same age ranges and separate them by gender, one employing the EWGSOP2 algorithm and the other the AWGS2. As in the previous case, for all age and gender groups, the prevalence was higher when the Asian criterion was employed, except for men in the 70–74 age group, where the prevalence was 8.1% higher when the European criterion is used. The most significant difference in prevalence was found in the older age group (75–79 years), where men and women presented prevalences 18.5% and 11.9% higher, respectively.

In the context of diagnosing severe sarcopenia, both EWGSOP2 and AWGS2 use physical performance as a parameter. The most employed test to assess this variable is walking speed, which can be measured over distances like 4 m or 6 m [53]. Physical performance correlates strongly with health and is therefore considered to be the most reliable indicator in clinical and research environments [55]. The EWGSOP2 includes tests for the evaluation of physical performance, the TUG and SPPB, both of which are related to mortality [57,58]. AWGS2 uses the 6 m test, SPPB, and 5-time chair stand as assessment tools for evaluating performance. While these tests share similarities, EWGSOP2 requires all of them to yield positive results for diagnosing severe sarcopenia, whereas AWGS2 requires only one [54].

In analyses of severe sarcopenia prevalence, the use of AWGS2 (9.95%) yields a higher rate compared to EWGSOP2 (1.8%).

Therefore, the variations in diagnostic algorithms developed by different working groups make it challenging to compare sarcopenia and severe sarcopenia prevalence across diverse populations. Limitations arise from differences in diagnostic criteria, assessment tools, tests used, and subject characteristics based on race.

## 5. Conclusions

In conclusion, the prevalence of sarcopenia varies according to the diagnostic algorithm used. However, the prevalence increases with advancing age and is higher in men in all cases. The most used diagnostic algorithms are the AWGS2 and the EWGSOP2, which involve different case search methods (AWGS2: SARC-F, SARC-CalF, calf circumference, pathologies, age; EWGSOP2: only SARC-F) but use similar tests (AWGS2: grip strength test, 5-time chair-stand test, gait speed; EWGSOP2: grip strength test, 5-time chair-stand test). However, the latter requires all tests to be positive for the diagnosis of sarcopenia. AWGS2 provides more options to advance to the next step of the diagnostic algorithm, while EWGSOP2 in many cases leaves positive diagnoses undetected, or yields a lower prevalence of sarcopenia. The diagnostic criterion with the highest prevalence of sarcopenia and severe sarcopenia is AWGS2. These differences in prevalence may be because each working group establishes different assessment tools and cut-off points. In sarcopenia case-finding, the substantial differences in sarcopenia prevalence rates underline the need for a uniform diagnostic standard that allows early detection of the disease and mitigates associated consequences, considering the anthropometric characteristics of different ethnicities.

## Figures and Tables

**Table 1:** Diagnostic algorithm and cut-off points for sarcopenia and severe sarcopenia according to different working groups.

	Variables/Test	AWGS1	AWGS2	EWGSOP1	EWGSOP2	FNIH	SDOC	IWGS
Case search	Questionnaire (SARC-F)	-	≥4 points	-	≥4 points	-		
	Questionnaire (SARC-CalF)	-	≥11 points	-	-	-		
	Calf circumference	-	M < 34 cm F < 33 cm	-	-	-		
	Pathologies	-	+	-	-	Weakness		
	Age	>60–65 years	-	>65 years	-	-		≥65 years old
Evaluation	Grip strength	M < 26 kg F < 18 kg	M < 28 kg F < 18 kg	M < 30 kg F < 20 kg	M < 27 kg F < 16 kg	M < 26 kg F < 16 kg	M< 35.5 Kg F < 20 kg	
	5-time chair stand	-	t ≥ 12 s	-	t > 15 s	-		
	Test 4 m	-	v ≤ 0.8 m/s	-	-	-	v < 0.8 m/s	v < 1 m/s
Confirmation	MMA				M < 20 kg F < 15 kg	M < 19.75 kg F < 15.02 kg		
	MMA/h^2^	BIA M < 7 kg/m^2^ F < 5.7 kg/m^2^	DEXA M < 7 kg/m^2^ F < 5.4 kg/m^2^ BIA M < 7 kg/m^2^ F < 5.7 kg/m^2^	DEXA or BIA M < 7.26 kg/m^2^ F < 5.45 kg/m^2^	DEXA or BIA M < 7 kg/m^2^ F < 6 kg/m^2^			DEXA M ≤ 7.23 kg/m^2^ F ≤ 5.57 kg/m^2^
	MMA/IMC^2^	-	-	-	-	M < 0.789 F < 0.512		
Severity	Test 4 m	-	-	v < 0.8 m/s	v ≤ 0.8 m/s	-		
	Test 6 m	v < 0.8 m/s	v < 1 m/s	-	-	-		
	Chair test	-	t ≥ 12 s	-	-	-		
	TUG	-	-	-	t ≥ 20 s	-		
	SPPB	-	≤9 points	-	≤8 points	-		
Confirmed diagnosis of sarcopenia		↓ Grip strength or ↓ walking speed + MMA/h^2^	Positive case search + ↓ MMA or↓ Physical performance	↓ MM + ↓ Grip strength or ↓ walking speed	SARC-F ≥ 4 points + ↓ Grip strength and saddle test + ↓ MM	↓ Grip strength + ↓ MMA/IMC^2^ or MMA	↓ Grip strength + ↓ Gait speed	↓ MMA/h^2^ + ↓ Gait speed
Diagnosis of severe sarcopenia		-	Positive case search + ↓ MMA + ↓ Physical performance (chair test)	↓ MM + ↓ Grip strength + ↓ walking speed	Sarcopenia + ↓ Physical performance	-	-	-

MM: Muscle Mass; MMA: Appendicular Muscle Mass; h: height; TUG: Timed Up and Go; SPPB: Short Physical Performance Battery; DEXA: Dual-energy X-ray Absorptiometry; BIA Bioelectrical Impedance Analysis; v: velocity; t: time; SARC-F (Strength, Assistance in walking, Rise from chair, Climb chair and Falls), SARC-CalF (Strength, Assistance in walking, Rise from chair, Climb chair, Falls and Calf); M: male; F: female; AWGS: Asian Working Group for Sarcopenia; EWGSOP: European Working Group on Sarcopenia in Older People; FNIH: Foundation for the National Institutes of Health; SDOC: Sarcopenia Definitions and Outcomes Consortium; +: plus; ↓: low.

**Table 2:** This table shows the combinations of thesaurus and natural terms used on the PUBMED search string and their corresponding results.

	Address	27 November 2023
Search Strategy	Natural Terms, MeSH and Equations	Results Obtained
#1	Sarcopenia (MeSH)	
#2	Diagnosis (MeSH) OR Consensus (MeSH) OR prevalence (MeSH)	
#3	Aged (MeSH) OR Aging (MeSH)	
#4	#1 AND #2 AND #3	4743
#5	Assessment (tiab) OR Older (tiab)	
#6	#4 AND #5	672
	Total, sum of the blocks #4 OR #6	5415

**Table 3:** List of the different working groups.

Working Groups	
Acronym	Name (year)
AWGS1	Asian Working Group for Sarcopenia (2010)
AWGS2	Asian Working Group for Sarcopenia (2014)
EWGSOP1	European Working Group on Sarcopenia in Older People 1 (2014)
EWGSOP2	European Working Group on Sarcopenia in Older People 2 (2019)
FNIH	Foundation for the National Institutes of Health (2014).
SDOC	Sarcopenia Definitions and Outcomes Consortium (2020)

**Table 4:** Summary of relevant findings from the 19 included articles.

Studies	Subjects	Ethnicity	Diagnosis	Outcomes
[21]	*n* = 4866 Male: 2446 Female: 2420 Age (60–70 years old): 3456 Age (70–80 years old): 1198 Age (≥80 years old): 212 Mean age: 67.7 ± 6.4 years old	Chinese	AWGS2	Probable sarcopenia prevalence by sex (%): Males: 46% Females: 40.8% Total: 43.4%
[22]	*n* (2012): 1371 *n* (2017): 1597 Male: 1307 Female: 1661 Age media: 74.2 ± 6.5 Age (65–69 years old): 898 Age (70–74 years old): 793 Age (75–79 years old): 619 Age (80–84 years old): 416 Age (≥85 years old): 242	Japanese	AWGS1	Sarcopenia prevalence by age, 2012 (%): 65–69 years old: 1.8% 70–74 years old: 4.8% 75–79 years old: 8.1% 80–84 years old: 10.4% ≥85 years old: 28.8% Sarcopenia prevalence by age, 2017 (%): 65–69 years old: 1.2% 70–74 years old: 3.1% 75–79 years old: 5.6% 80–84 years old:15.7% ≥85 years old: 26% Difference in prevalence according to age and sex 2012 (%): 65–69 years old: ↑ 0.9% males 70–74 years old: ↑ 2.1% females 75–79 years old: ↑ 3.9% females 80–84 years old: ↑ 1% females ≥85 years old: ↑ 9.4% females Difference in prevalence according to age and sex 2017 (%): 65–69 years old: ↑ 1.4% females 70–74 years old: ↑ 0.8% females 75–79 years old: ↑ 3% females 80–84 years old: ↑ 4.8% females ≥85 years old: ↑ 1.6% females
[23]	*n* = 2129 Male: 1177 Female: 1148 Mean age: 76.76 ± 3.7 years old	Korean	AWGS2	Sarcopenia prevalence by age (%): 70–74 years old: 13.8% 75–79 years old: 23% 80–84 years old: 38% Difference in prevalence in men by age (%): Total: 17% 70–74 years old: 11.6% 75–79 years old: 28.5% 80–84 years old: 45.6% Difference in prevalence in women by age (%): Total: 9.9% 70–74 years old: 15.6% 75–79 years old: 16.9% 80–84 years old: 27.9%
[24]	*n* = 9170 males Age: ≥65 years old Mean age: 74.3 ± 4.9 years old	American, Sweden, English and Belgian	EWGSOP2 SDOC	Sarcopenia prevalence according to EWGSOP2 by age (%): Total: 1.1% 64–70 years old: 0.3% 71–74 years old: 0.6% 75–70 years old: 1% 80–100 years old: 4.8% Sarcopenia prevalence according to SDOC by age (%): Total: 1.7% 64–70 years old: 0.4% 71–74 years old: 1.8% 75–79 years old: 2.1% 80–100 years old: 5.1%
[25]	*n* = 631 Male: 213 Female: 418 Age: 65–89 years old Mean age: 73.5 ± 7.7 years old	Chinese	AWGS1	Sarcopenia prevalence by age in men (%): Total: S:12.2%, SO:7%, T:19.2% 65–69 years old: S:5.9%, SO:2.9%, T:8.8% 70–74 years old: S:12.5%, SO:6.3%, T:18.8% 75–79 years old: S:13.6%, SO:13.5%, T:27.1% ≥80 years old: S:21.6%, SO:13.5%, T:35.1% Sarcopenia prevalence by age in women (%): Total: S:6.2%, SO 2.4%, T8.6% 65–69 years old: S:3.1%, SO 0.8%, T3.9% 70–74 years old: S:3.5%, SO 1.8%, T5.3% 75–79 years old: S:10.3%, SO:3.1%, T13.4% ≥80 years old: S:10.3%, SO:5.1%, T15.4%
[26]	*n* = 1105 Male: 557 Female: 548 Age: 60–89 years old Mean age 71.67 ± 0.31 years old	Chinese	AWGS2	Sarcopenia prevalence (%): Men: 12.93% Women: 21.72% Total: 17.29%
[27]	*n* = 310 Male: 89 Female:221 Age: 65–91 years old Mean age: 76 ± 5.8 years old	Japanese	EWGSOP2	Sarcopenia prevalence by age in men (%): Total: 10.1% 65–69 years old: 0% 70–74 years old:15.5% 75–79 years old: 3% 80–84 years old: 12.5% 85–91 years old: 22.5% Sarcopenia prevalence by age in women (%): Total: 7.2% 65–69 years old: 2.5% 70–74 years old:6.5% 75–79 years old: 11% 80–84 years old: 6% 85–91 years old: 13.5%
[28]	*n* = 745 Male: 221 Female: 524 Age: ≥65 years old Mean age: 76.6 ± 6.9 years old	Brazilian	EWGSOP 1	Sarcopenia prevalence by sex (%): Men: 9% Women:8% Total: 10.74% Sarcopenia prevalence by age (%): 65–74 years old: 6.3% 75–84 years old: 48.8% >85 years old: 45% Severe sarcopenia prevalence by sex (%): Men: 6.9% Women: 10.9%
[29]	*n* = 1082 Male: 466 Female: 616 Age: ≥60 years old Mean age: 76.6 ± 7.11 years old	Japanese	AWGS 2	Probable sarcopenia in men by age (%): Total: 52.79% 60–74 years old: 26.18% ≥75 years old: 36.05% Probable sarcopenia in women by age (%): Total: 44.48% 60–74 years old: 13.31% ≥75 years old: 31.16%
[30]	*n* = 2099 Male: 1053 Female: 1046 Age: 70–84 years old Mean age 75.9 ± 4 years old	Korean	EWGSOP2 AWGS1	Sarcopenia prevalence according to EWGSOP2 (%): Men: 11.9% Women: 6.7% Total: 9.3% Sarcopenia prevalence according to AWGS1 (%) Men: 10.2% Women: 8% Total: 9.1% Severe sarcopenia prevalence according to EWGSOP2 (%): Men: 1.9% Women: 1.6% Total: 1.8%
[31]	*n* = 388 Male: 134 Female: 254 Age: ≥65 years old Mean age: 77.8 ± 6.26 years old	Korean	AGWS2	Sarcopenia prevalence according to grip strength: 37.63% Sarcopenia prevalence according to gait speed: 40.97%
[32]	*n* = 1040 Male: 693 Female: 347 Age: ≥60 years old Mean age: 71.4 ± 7.52 years old	Chinese	AWGS2	Sarcopenia prevalence by age (%): 60–69 years old: 19.5% 70–79 years old: 28.9% ≥80 years old: 51.5% Sarcopenia prevalence by sex (%): Men: 26.2% Women: 25.2% Total:27.1%
[33]	*n* = 892 Male: 278 Female: 614 Age: ≥60 years old Mean age: 70 ± 4.5 years old	Thai	AWGS2	Sarcopenia prevalence (%): Total: 22.2% Male: 24.1% Women: 21.3% Severe sarcopenia prevalence (%): Total: 9.4% Male: 11.5% Women: 8.5%
[34]	*n* = 161 females Age: ≥65 years old Mean age: 74.4 ± 7.3 years old	Brazilian	EWGSOP2	Sarcopenia prevalence: ↓ MM and grip strength: 2.7% ↓ MM and chair test: 6.5% Severe sarcopenia prevalence: ↓ MM, HGS and GS: 3.4% ↓ MM, HGS and TUG: 5.4% ↓ MM, HGS and SPPB: 1.4%
[35]	*n* = 1407 Male: 581 Female: 826 Age: ≥65 years old Mean age: 71.91 ± 5.59 years old	Chinese	AWGS 2	Sarcopenia prevalence by sex (%): Total: S:9.74%, SO:9.95%, T:19.69% Men: S:9.29%, SO:13.94%, T:23.23% Women: S:10.05%, SO:7.14%, T:17.19% Sarcopenia prevalence by age (%): 65–69 years old: S:7.69%, SO:4.37%, T:12.06% 70–74 years old: S:9.17, SO:8.49%, T:17.66% 75–79 years old: S:11.97%, SO:14.53, T:26.5% ≥80 years old: S:15.15%, SO:26.67%, T:41.82% Sarcopenia prevalence in women by age (%): 65–69 years old: S:7.36%, SO:3%, T:10.36% 70–74 years old: S:9.68, SO:6.45%, T:16.13% 75–79 years old: S:12.3%, SO:11.48%, T:23.78% ≥80 years old: S:19.1%, SO:20.22%, T:39.32% Sarcopenia prevalence in men by age (%): 65–69 years old: S:8.29, SO:6.83, T:15.12% 70–74 years old: S:8.51, SO:11.17%, T:19.68% 75–79 years old: S:11.61, SO:17.86%, T:29.47% ≥80 years old: S:10.53%, SO:34.21%, T:44.74%
[36]	*n* = 1287 Male: 505 Female: 782 Age: 75–84 years old Mean age: 77.9 ± 1.92 years old	Korean	FNIH	Sarcopenia prevalence by sex (%): Male: 39.41% Women: 36.45% Total: 37.43%
[37]	*n* = 1851 Male: 917 Female: 934 Age: ≥65 years old Mean age: 74.15 ± 5.98 years old	Japanese	AWGS 2	Sarcopenia prevalence in men by age (%): Total: 11.5% 65–69 years old: 3.7% 70–74 years old: 7.4% 75–79 years old: 21.5% ≥80 years old: 32.5% Sarcopenia prevalence in women by age (%): Total: 16.7% 65–69 years old: 6.6% 70–74 years old: 12.7% 75–79 years old: 22.9% ≥80 years old: 47.7%
[38]	*n* = 665 Male: 342 Female: 323 Age: 60–96 years old Mean age 70 ± 5.55 years old	Australian	EWGSOP 2	Sarcopenia prevalence in men by age (%): Total: 2.9% 60–69 years old: 0% 70–79 years old:2.6% ≥80 years old: 11.1% Sarcopenia prevalence in women by age (%): Total: 0.9% 60–69 years old: 0% 70–79 years old: 1.6% ≥80 years old: 21.1%
[39]	*n* = 719 Male: 282 Female: 437 Mean age: 85.5 ± 0.4 years old	American	EWGSOP 1	Sarcopenia prevalence by sex (%): Men: 20.91% Women: 20.59% Total: 20.8%

AWGS: Asian Working Group for Sarcopenia; EWGSOP: European Working Group on Sarcopenia in Older People; FNIH: Foundation for the National Institutes of Health; SDOC: Sarcopenia Definitions and Outcomes Consortium; MM: muscle mass; TUG: Timed Up and Go; SPPB: Short Physical Performance Battery; N: all subjects; S: sarcopenia; SO: sarcopenic obesity; T: total; HGS: handgrip strength; GS: gait speed; ↑: increase; ↓: low.

**Table 5:** Mean prevalence of sarcopenia according to age and sex for each diagnostic algorithm.

Group	Sex	Age				
		65–69	70–74	75–79	80–84	>85
AWGS1	Men	8.8%	18.8%	27.1%	35.1%	-
	Women	3.9%	5.3%	13.4%	15.4%	-
AWGS2	Men	7.85%	13.52%	25.49%	38.62%	-
	Women	8.48%	14.42%	23.34%	43.51%	-
EWGSOP1	Men	6.3%		48.8%		55.67%
	Women					
EWGSOP2	Men	-	10.55%		11.8%	22.5%
	Women	2.5%	9.55%		13.55%	13.5%
SDOC	Men	0.4%	1.8%	2.1%	5.1%	-
	Women					
Mean	Men	5.55%	16.6%	26.3%	36.86%	
	Women	4.96%	9.86%	18.37%	29.46%	
	Total	5.32%	10.77%	18.29%	23.3%	27%

**Table 6:** Methodological evaluation using the modified NOS scale.

Type of Study		NOS (Modified)						
		1	2	3	4	5	6	Score
Chen et al., 2022 [21]	Cross-sectional study	*	*	*	*	**	***	8/10
Nakamura et al., 2021 [22]	Cross-sectional study	*	*	*	**	*	***	9/10
Shin et al., 2022 [23]	Cross-sectional study	*	*	*	**	**	***	10/10
Westbury et al., 2023 [24]	Cross-sectional study	*	*	*	**	**	**	9/10
Du et al., 2019 [25]	Cross-sectional study	*		*	*	*	***	7/10
Yang et al., 2022 [26]	Cross-sectional study	*		*	**	*	***	8/10
Su et al., 2019 [27]	Cross-sectional study	*	*	*	*	**	***	9/10
Moreira et al., 2019 [28]	Cross-sectional study	*			*		***	5/10
Yao et al., 2022 [29]	Cross-sectional study	*	*	*	*	**	***	10/10
Kim et al., 2019 [30]	Cross-sectional study	*		*		*	***	6/10
Chang et al., 2020 [31]	Cross-sectional study	*		*	*	*	**	6/10
Zhong et al., 2022 [32]	Cross-sectional study	*	*	*	*	**	***	9/10
Sri-on et al., 2022 [33]	Cross-sectional study	*			**	*	***	7/10
Sutil et al., 2023 [34]	Cross-sectional study	*	*		*	*	***	7/10
Lu et al., 2022 [35]	Cross-sectional study	*			*	**	***	7/10
Hwang et al., 2022 [36]	Cross-sectional study			*	**	*	***	7/10
Kitamura et al., 2021 [37]	Cross-sectional study	*		*	*	**	***	8/10
Sui et al., 2021 [38]	Cross-sectional study	*		*	**	**	***	9/10
Dodds et al., 2023 [39]	Cross-sectional study	*	*		**	*	***	8/10

*: 1 point; **: 2 points and ***: 3 points of score.

## Data Availability

Not applicable.
